# Multiple risk factor intervention reduces carotid atherosclerosis in patients with type 2 diabetes

**DOI:** 10.1186/1475-2840-13-95

**Published:** 2014-05-23

**Authors:** Norbert J Tripolt, Sophie H Narath, Michaela Eder, Thomas R Pieber, Thomas C Wascher, Harald Sourij

**Affiliations:** 1 Department for Internal Medicine, Division of Endocrinology and Metabolism, Medical University of Graz, Graz, Austria; 2 Cardiovascular Diabetology Research Group, Medical University of Graz, Graz, Austria; 3 Institute for Biomedicine and Health Sciences, Joanneum Research, Graz, Austria; 4 1st Medical Department, Hanusch Hospital, Vienna, Austria

**Keywords:** Intensified risk factor intervention, Carotid intima media thickness, Type 2 diabetes, Cardiovascular surrogate measurements, Carotid atherosclerosis

## Abstract

**Background:**

Patients with rapid progression of carotid intima media thickness (CIMT) were shown to have a higher future risk for cardiovascular events.

The aim of this study was to investigate the impact of multiple risk factor intervention on CIMT progression and to establish whether new cardiovascular surrogate measurements would allow prediction of CIMT changes.

**Materials and methods:**

In this prospective, open, 2-years study, we included 97 patients with type 2 diabetes and at least two insufficiently treated cardiovascular risk factors, i.e. HbA_1c_ > 7.5% (58 mmol/mol); LDL-cholesterol >3.1 mmol/l or blood pressure >140/90 mmHg. Treatment was intensified according to current guidelines over 3 months with the aim to maintain intensification over 2 years.

The primary outcome was the change in CIMT after 2 years. We also assessed markers of mechanical and biochemical endothelial function and endothelial progenitor cells before and after 3 months of treatment intensification. For testing differences between before and after multifactorial treatment measurements we used either the paired student’s *t*-test or the Wilcoxon signed-rank test, depending on the distribution of the data. Additional, explorative statistical data analysis was done on CIMT progression building a linear multivariate regression model.

**Results:**

Blood glucose, lipids and blood pressure significantly improved during the first 3 months of intensified treatment, which was sustained over the 2-year study duration. Mean CIMT significantly decreased from baseline to 2 year (0.883 ± 0.120 mm vs. 0.860 ± 0.130 mm; p = 0.021). None of the investigated surrogate measures, however, was able to predict changes in IMT early after treatment intensification.

**Conclusions:**

Intensification of risk factor intervention in type 2 diabetes results in CIMT regression over a period of 2 years. None of the biomarkers used including endothelial function parameters or endothelial progenitor cells turned out to be useful to predict CIMT changes.

**Trial registration:**

Clinical Trial Registration – Unique identifier: NCT00660790

## Introduction

Patients with type 2 diabetes face a significantly increased risk for cardiovascular events as well as mortality compared to subjects without diabetes [1]. The STENO-2 study was the first study which impressively demonstrated a halving of the risk for cardiovascular events and mortality by target driven, intensified, multifactorial risk factor intervention compared to conventional, less stringent risk factor management [2].

However, epidemiologic data suggest that the cardiovascular risk attributable to type 2 diabetes remains about two-fold increased even after adjustment for established risk factors such as hyperglycaemia, hypertension or hyperlipidaemia [3]. Therefore it would be crucial to identify those subjects with the highest residual risk in order to intensify preventive therapies.

Common carotid artery intima-media thickness (CIMT), measured by B-mode ultrasound, is a validated surrogate measure of preclinical atherosclerosis and was shown to be a predictor of future cardiovascular events [4]. Recently, in particular rapid progression of CIMT has been associated with adverse cardiovascular outcome [5].

In addition, CIMT can be assessed quickly, non-invasively, and inexpensively with high-resolution ultrasound and therefore changes in CIMT might be a useful early measurement of treatment response.

Previous data have shown that statin treatment [6,7] or blood pressure treatment [8] are able to reduce CIMT progression, however, whether or not multifactorial risk factor intervention as recommended by current guidelines is able to reduce CIMT progression in subjects with type 2 diabetes, has not been investigated yet.

We have performed a study in patients with type 2 diabetes with inadequately controlled cardiovascular risk factors and have intensified their treatment according to current treatment guidelines over a 3 months period and aimed to maintain this intensification over 2 years. The primary aim was to investigate the impact of this intervention on CIMT during these 2 years. The secondary aim was to investigate whether the treatment intensification has an impact on novel cardiovascular surrogate measurements such as the number of endothelial progenitor cells, endothelial function or selected biomarkers and if so, whether they prove clinically useful as early predictors for CIMT changes after 2 years.

## Materials and methods

### Study design

We performed a single-center, prospective, open, 2-year clinical trial. At baseline, after 3 months and after 2 years patients were seen at the Division of Endocrinology and Metabolism at the Medical University of Graz, Austria for a detailed examination. Examinations were conducted in the morning following an overnight fast. The subject enrollment started in March 2008 and ended in May 2010. Data were first collected on a paper case report form and were then transferred to electronic case report forms.

Reporting the study conforms to STROBE along with references to STROBE and the broader EQUATOR guidelines [9].

Patients received a target oriented, intensified treatment of risk factors according to current national treatment guidelines (available at http://www.oedg.org). All patients received personalized lifestyle counseling. After 3 months all modifiable risk factors were re-evaluated. Between the 3 months visit and the 2 year follow up all patients were regularly followed by their usual care provider and received regular phone calls by the study site to discuss risk factor control and the usual care provider was reminded of treatment targets if necessary in order to maintain adequate cardiovascular risk factor control. If risk factor management remained unsatisfactory, the patient was invited for a follow-up visit at the diabetes outpatient clinics of the Medical University of Graz. 24 months after inclusion into the study CIMT was measured again.

### Participants

Patients with type 2 diabetes mellitus without previous vascular events were identified from the outpatient clinic at the Division of Endocrinology and Metabolism at the Medical University of Graz. Written informed consent was obtained from all subjects.

Patients were eligible if they were 45 to 75 year old, had type 2 diabetes mellitus with at least two insufficiently controlled risk factors: i.e.: LDL-cholesterol >3.1 mmol/l, blood pressure >140/90 mmHg (either or), HbA_1c_ >7.5% (>58 mmol/mol), all listed parameters could be treated or untreated. Diabetes mellitus was defined as fasting blood glucose ≥7.0 mmol/l or a history of established diabetes according to WHO criteria [10]. Patients with a history of any cardiovascular events, heart failure (>NYHA II), serum creatinine >265.2 μmol/L, aspartat-aminotransferase/alanine-aminotransferase >3-fold ULN of the reference range and major psychiatric disorders were excluded.

### Primary outcome measurement

The primary outcome was the change in CIMT from baseline to 2 years. High-resolution carotid artery ultrasound measurements were obtained using a portable ultrasound Acuson Cypress (Siemens Medical Solutions USA Inc., Mountainview, California). All recordings were performed by a single, certified sonographer who had no access to the baseline measurements at the time of follow up assessment. The common carotid artery, carotid bulb, and internal as well as external arteries were examined. CIMT was measured in the common carotid artery 5 mm distal to the bifurcation over a length of 10 mm. Digital images were electronically transferred to an offline workstation where as a first step an automated quality index of the image was assessed and if the predefined quality criteria were met, CIMT measurements were performed by using a dedicated analysis software (M’Ath, Metris SRL, Argenteuil, France) in order to reduce measurement variability. Mean CIMT was calculated as the average of multiple measurements of the far wall IMT of the left and right common carotid artery.

A 75 g oral glucose tolerance test was performed in all subjects after a 12-h overnight fast at baseline and after 3 months.

### Reactive hyperaemia index (RHI)

Endo-PAT 2000 (Itamar Medical Ltd., Caesarea, Israel) was used to measure endothelium-dependent vaso-reactivity as previously described [11].

### Endothelial progenitor cells (EPCs)

Peripheral white blood cells were analysed for the expression of endothelial progenitor cell characterizing surface markers by using flow cytometry as previously described [12].

### Symmetric dimethylarginine (SDMA) and asymmetric dimethylarginine (ADMA)

We measured SDMA and ADMA in frozen EDTA plasma (-20°C) by high-performance liquid chromatography (HPLC) with solid phase extraction and precolumn derivatization [13]. Within-day CVs for SDMA were 4.6% (0.60 μmol/L) and 1.9% (1.0 μmol/L), and between-day CVs were 9.8% (0.60 μmol/L) and 6.1% (1.0 μmol/L). Within-day CVs for ADMA were 3.1% (0.62 μmol/L) and 1.0% (2.0 μmol/L), and between-day CVs were 9% (0.62 μmol/L) and 1.5% (2.0 μmol/L).

### High sensitivity c-reactive protein (hs-CRP)

Hs-CRP was measured with a particle-enhanced immunoturbimetric assay (Roche Diagnostics GmbH, Mannheim, Germany), with a detection limit of 0.1 mg/l.

### PROCAM (PROspective cardiovascular Münster) risk score

The PROCAM-algorithm estimates the risk for acute coronary events (myocardial infarction, sudden cardiac death) within 10 years. The calibrated risk score includes: age, LDL- cholesterol, smoking, HDL-cholesterol, systolic blood pressure, family history of premature myocardial infarction, diabetes mellitus, and triglycerides [14].

### UKPDS risk engine

The UKPDS Risk Engine 2.0 is a type 2 diabetes specific risk calculator based on 53,000 patient years of data from the UK Prospective Diabetes Study. The UKPDS risk engine provides risk estimates and 95% confidence intervals, in individuals with type 2 diabetes not known to have heart disease, for non-fatal and fatal coronary heart disease, for fatal coronary heart disease, for non-fatal and fatal stroke as well as for fatal stroke. These can be calculated for any given duration of type 2 diabetes based on current age, sex, ethnicity, smoking status, presence or absence of atrial fibrillation and levels of HbA1c, systolic blood pressure, total cholesterol and HDL cholesterol [15].

### Glucose lowering

Patients were treated according to current international treatment guidelines for type 2 diabetes mellitus at this time. The generally recommended treatment target for HbA_1c_ was ≤6.5% (≤48 mmol/mol) with a personalized treatment target approach. Thus, the first-line therapy was metformin followed by a second oral antihyperglycaemic agent (AHA) and extended to a triple oral therapy if required. If no sufficient glycaemic control was achieved with oral AHAs, insulin therapy was added. If HbA_1c_ was >8.0% (>64 mmol/mol) at the baseline visit while the patient was on no or one oral AHA only, treatment was intensified by commencing two additional AHAs simultaneously.

### Lipid treatment

The LDL-cholesterol target was <2.6 mmol/l in all patients included. A statin was used as first-line medication and the dose escalated if required. Another lipid lowering agent was added if the LDL-cholesterol target was not reached within 3 months.

### Blood pressure treatment

A blood pressure of ≤130/80 mmHg (preferably serial home measurements if available, otherwise office reading) was the treatment target. First-line therapy was targeting the renin-angiotensin-system (i.e. angiotensin converting enzyme inhibitors or angiotensin receptor blocker). Further agents were added if required.

### Statistical analysis

The distribution of the continuous variables was evaluated by a Kolmogorov-Smirnov test. The paired student’s *t*-test or the Wilcoxon signed-rank test was used to compare the measurements before and after multifactorial treatment, as appropriate, depending on the distribution of the data.

Linear mixed effects models were built with time in months as influence variable (fixed effect) and patient-ID as random effect to estimate trend over time for selected risk factors to deal with the limited number of time points available.

Chi-square test was used to compare categorical variables. Correlations were made using a Pearson’s product moment correlation coefficient.

A two-tailed p-value of less than 0.05 was considered as statistically significant.

Additional, explorative statistical data analysis was done building a linear multivariate regression model on the progression of CIMT. Variable selection for influence-variables to predict CIMT progression was based on univariate regression modelling. Baseline values from candidate variables and changes in these values from baseline to 3-months with a p-value below 0.2 together with age and sex (as potential confounder variables) were included as influence variables into the linear multivariate regression model with CIMT progression (ΔCIMT) as outcome variable. Amongst others the following candidate variables were included for the linear multivariate regression model: CIMT, triglycerides, coronary heart disease (CHD) risk score, PROCAM risk score, waist circumference, HDL-cholesterol, serum creatinine, 2h-glucose, smoking, statin therapy – all measured at baseline as well as changes in triglycerides NTproBNP, HDL-cholesterol, 2h-glucose from baseline to 3 months as well as statin therapy after 3 months together with age and sex (as potential confounder variables) (see the `List of parameters entering univariate analysis´ section) were included into the multivariate regression analysis with ΔCIMT as outcome variable. The Model selection process for multivariate modelling (which exposure variables to include in the final model) was based on the Akaike Information Criterion using backward selection. If one or more variables were highly correlated, the less significant ones were excluded; the variance inflation factor was used as a criterion for collinearity, with a cut off < 3. Only complete cases for candidate variables were included into modelling.

### List of parameters entering univariate analysis

Asymmetric dimethylarginine (Baseline), Asymmetric dimethylarginine (Δ), Age, Alanin-Aminotransferase (Baseline), Alanin-Aminotransferase (Δ), Apolipoprotein B (Baseline), Apolipoprotein B (Δ), Body mass index (Baseline), Body mass index (Δ), CD34CD133 (Baseline), CD34CD133 (Δ), Coronary heart disease UKPDS risk engine 2.0 (Baseline), Coronary heart disease UKPDS risk engine 2.0 (Δ), Diabetes duration (Baseline), Global arginine bioavailability ratio (Baseline), Global arginine bioavailability ratio (Δ), Gamma Glutamyltransferase (Baseline), Gamma Glutamyltransferase (Δ), Fasting blood glucose (Baseline), Fasting blood glucose (Δ), Blood glucose 2h (Baseline), Blood glucose 2h (Δ), urinary albumin (Baseline), Urinary albumin (Δ), Hb_A1c_ (Baseline), Hb_A1c_ (Δ), HDL-cholesterol (Baseline), HDL-cholesterol (Δ), HOMA index (Baseline), HOMA index (Δ), Intima media thickness (Baseline), Fasting insulin (Baseline), Fasting insulin (Δ), Serum creatinine (Baseline), Serum creatinine (Δ), LDL-cholesterol (Baseline), LDL-cholesterol (Δ), Lipoprotein (a) (Baseline), NTproBNP (Baseline), NTproBNP (Δ), Reactive hyperaemia index (Baseline), reactive hyperaemia index (Δ), Blood pressure diastolic (Baseline), Blood pressure diastolic (Δ), Blood pressure systolic (Baseline), Blood pressure systolic (Δ), Sex, Smoking (Baseline), Triglycerides (Baseline), Triglycerides (Δ), 25-OH-vitamin d3 (Baseline), 25-OH-vitamin d3, Waist circumference (Baseline).

Explained variance of the multivariate model above 30% and p-value <0.05 were taken to be predictive powerful and useful to interpret.

Univariate and multivariate linear modelling was done using the statistical software R version 2.15, whereas further statistical analyses were performed by using SPSS 19.0 software (SPSS Inc, Chicago, Ill).

## Results

### Patients

**Figure 1:**
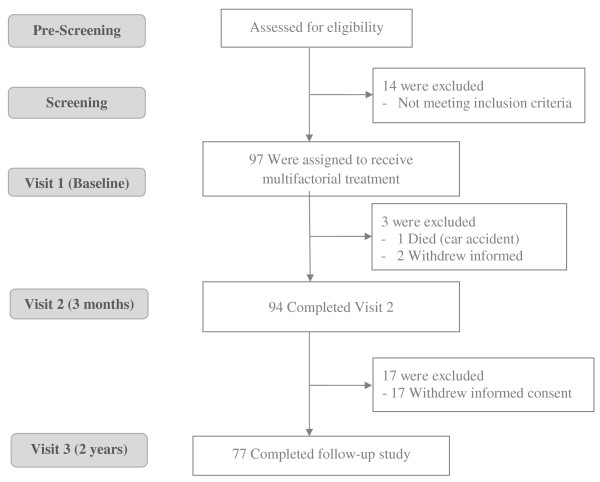
Study flow chart.

111 subjects were screened for the study between March 2008 and May 2010. Study inclusion criteria were met by 97 patients who were enrolled in the study. (For patient flow chart see Figure 1). Baseline characteristics of completers and non-completers of the study are presented in Table 1. Non-completers were more often females and smokers, were older and had slightly higher Hb_A1c_ values, but did not differ otherwise from those who have completed the study.

**Table 1:** Baseline-characteristics compared completers and non-completers of the study

	**Completers (n = 77)**	**Non-completers (n = 20)**	**p-value**
Sex (female/male)	27/50	9/11	0.001
Age (years)	60 ± 8	65 ± 10	0.048
Height (cm)	170 ± 9	167 ± 9	0.061
Weight (kg)	91 ± 15	91 ± 18	0.989
Smokers	25 (25.8%)	8 (40%)	0.002
Packyears (years)	31 (12 – 56)	26 (10 -58)	0.599
Hip circumference (cm)	113 ± 9	112 ± 9	0.717
Waist circumference (cm)	109 ± 12	107 ± 13	0.396
Blood pressure systolic (mmHg)	154 ± 17	146 ± 19	0.138
Blood pressure diastolic (mmHg)	90 ± 9	88 ± 11	0.473
Duration of diabetes mellitus (years)	7.7 ± 6.8	7.5 ± 5.4	0.897
Hb_A1c_ % (mmol/mol)	8.2 ± 1.1% (66 ± 12)	8.6 ± 0.7% (70 ± 8)	0.049
Total cholesterol (mmol/l)	4.93 ± 1.11	5.02 ± 1.11	0.785
Low density lipoproteins (mmol/l)	2.79 ± 1.11	2.79 ± 1.12	0.937
High density lipoproteins (mmol/l)	1.14 ± 0.36	1.11 ± 0.46	0.738
CIMT (mm)	0.883 ± 0.120	0.875 ± 0.171	0.840
RHI	1.70 (1.46 – 2.08)	1.72 (1.38 – 2.07)	0.590
CD133 + VEGF-R2 (per 10^6^ lymphomonocytes)	15 (7 – 28)	14 (5 – 21)	0.514
ADMA (μM/L)	0.625 ± 0.080	0.606 ± 0.082	0.313
SDMA (μM/L)	0.562 ± 0.131	0.543 ± 0.110	0.445
Serum creatinine (μmol/l)	88.4 ± 26.5	84.0 ± 36.0	0.604
Alanin-Aminotransferase (U/l)	29 (21 – 40)	24 (21 – 39)	0.836
Gamma Glutamyltransferase (U/l)	35 (21 – 59)	28 (21 – 38)	0.824
Aspartat-Aminotransferase (U/l)	27 (21 – 36)	49 (28 – 91)	0.246

Data are presented as means ± standard deviation or, in the case of a skewed distribution, as medians (interquartile ranges). ADMA = asymmetric dimethylarginine; SDMA = symmetric dimethylarginine; HbA1c = glycated hemoglobin A1c; cIMT = common carotid artery intima-media thickness; RHI = Reactive hyperaemia Index.

Participants (38 f/59 m) were in mean 60 years old with mean diabetes duration of 7.7 years. An extensive list of baseline characteristics is provided in Tables 1 and 2.

**Table 2:** Changes in cardiovascular risk factors

	**Baseline (n = 97)**	**3 months (n = 94)**	**2 years (n = 77)**	**p-value (baseline to 3 months)**	**p-value (baseline to 2 years)**
***Traditional risk factors***					
Total cholesterol (mmol/l)	4.93 ± 1.11	4.28 ± 1.11	4.54 ± 1.29	<0.001	0.007
Low density lipoproteins (mmol/l)	2.79 ± 1.11	1.96 ± 1.01	2.40 ± 1.11	<0.001	0.008
High density lipoproteins (mmol/l)	1.14 ± 0.36	1.26 ± 0.34	1.24 ± 0.36	<0.001	0.001 ^*^
Triglycerides (mmol/l)	1.86	1.46	1.64	<0.001	0.205
	(1.31 – 2.66)	(1.06 – 2.23)	(1.27 -2.52)		
ApoA1 (g/l)	1.43 ± 0.22	1.46 ± 0.23	-	0.065	-
ApoB (g/l)	0.94 ± 0.23	0.81 ± 0.25	-	<0.001	-
Blood pressure systolic (mmHg)	154 ± 17	135 ± 12	139 ± 16	<0.001	<0.001 ^*^
Blood pressure diastolic (mmHg)	90 ± 9	82 ± 8	86 ± 16	<0.001	0.005
HbA_1c_ % (mmol/mol)	8.2 ± 1.1	7.2 ± 0.9	7.7 ± 1.1	<0.001	0.001
	(66 ± 12)	(54 ± 14)	(61 ± 12)		
Fasting blood glucose	9.9 ± 2.7	8.1 ± 2.3	-	<0.001	-
2h blood glucose (mmol/l)	18.2 ± 4.9	16.0 ± 4.5		<0.001	
BMI (kg/m^2^)	31.8 ± 4.7	31.4 ± 5.3	31.2 ± 4.6	0.465	0.807
***Biomarkers***					
RHI	1.70	1.59	-	0.072	-
	(1.46 – 2.08)	(1.38 – 1.94)			
CD133 + VEGF-R2 (per 10^6^ lymphomonocytes)	15	17	-	0.164	-
	(7 – 28)	(9-32)			
ADMA (μM/L)	0.625 ± 0.080	0.627 ± 0.075	-	0.835	-
SDMA (μM/L)	0.562 ± 0.131	0.599 ± 0.145	-	0.001	-
hsCRP (mg/L)	3.5	2.2	-	<0.001	-
	(2.1 – 5.9)	(1.2 – 4.1)			
NTproBNP (pg/mL)	104.8 ± 121.7	97.7 ± 103.6	-	0.412	-

Data are presented as means ± standard deviation or, in the case of a skewed distribution, as medians (interquartile ranges).

NTproBNP = N-terminal Pro B-Type Natriuretic Peptide; HbA_1c_ = glycated haemoglobin A1c; HOMA–IR = Homeostasis Model Assessment of Insulin Resistance; BMI = body mass index; ADMA = asymmetric dimethylarginine; SDMA = symmetric dimethylarginine; hsCRP = high sensitivity C-reactive protein; RHI = Reactive hyperaemia Index; *p-value for trend ≤0.05.

### Risk factor management

In order to treat risk factors to target, the use of oral AHAs, insulin, statins and blood pressure lowering medication was significantly increased over the 3 months period of treatment intensification. Detailed changes in pharmacological treatments are outlaid in Table 3. All targeted risk factors significantly improved from baseline to 3 months. Although an increase in all risk factor levels over the 21 months following the intensive treatment period was noted, the mean HbA_1c_, LDL-cholesterol and blood pressure levels at 24 months were still significantly lower than at the beginning of the study (Table 2). In addition, triglycerides improved from baseline to 3 months (p = 0.007), however, the reduction was not sustained until the 2 years follow up visit (p = 0.292).

**Table 3:** Changes in cardiovascular medication

	**Baseline (n = 97)**	**3 months (n = 94)**	**2 years (n = 77)**	**p-value (baseline - 3 months)**	**p-value (baseline - 2 years)**
***Glucose lowering treatment (%)***					
Metformin	62.9	75.5	67.1	0.001	0.655
Acarbose	3.1	2.1	1.2	0.320	0.317
Sulfonylureas	24.7	29.8	19.5	0.158	0.285
Glitazones	11.3	25.5	19.5	<0.001	0.058
DPP-IV Inhibitors	9.3	19.1	26.8	0.002	0.001
Insulin	26.8	32.3	29.3	0.027	0.105
Other OADs	3.1	3.3	2.4	1.000	0.564
***Lipid lowering treatment (%)***					
Statins	35.1	73.4	68.3	<0.001	<0.001
Ezetimibe	4.1	4.3	1.2	1.000	0.157
Fibrates	6.2	7.5	4.8	0.564	1.000
***Antihypertensive treatment (%)***					
ACE-Inhibitors	46.4	61.7	46.3	<0.001	1.000
Ca-channel blockers	21.6	47.9	46.3	<0.001	<0.001
Beta-blockers	35.1	40.4	42.7	0.102	0.109
AT_2_-blockers	21.6	28.7	30.0	0.033	0.132
Other Antihypertensives	37.1	46.8	42.5	0.020	0.516

OAD = oral antidiabetic drugs; DPP-IV = Dipeptidylpeptidase IV; ACE = angiotensin converting enzyme; AT_2_ = angiontensin-2-receptor antagonist; ca-channel = calcium-channel.

### Primary outcome

Multifactorial risk factor intervention was associated with a significant regression in mean CIMT from baseline to study end (0.883 ± 0.120 mm vs. 0.860 ± 0.130 mm; p = 0.021/relative reduction: 2.6%). 51 participants (66%) showed a regression or no measureable change of CIMT after 2 years, 26 (34%) progressed (0.061 ± 0.044 mm). There were no significant differences in baseline characteristics between patients with a regression and those with progression of CIMT after 2 years.

Mean CIMT significantly correlated with age at baseline (r = 0.365; p < 0.001), duration of diabetes (r = 0.273; p = 0.007) and systolic blood pressure (r = 0.320; p = 0.001). In addition mean CIMT significantly correlated with all UKPDS risk engine scores (p < 0.001 for all) and CIMT changes from baseline to 2 years correlated with changes in triglycerides (r = 0.292; p = 0.012).

### Endothelial function and endothelial progenitor cells

No associations between RHI and cardiovascular risk factors as well as changes in RHI and changes in risk factors were observed. Likewise no changes were observed in other measures of endothelial function such as ADMA and SDMA. We also assessed the number of CD133/VEGF-R2 positive cells in peripheral blood before and after the intervention. No changes in the number of endothelial progenitor cells were observed (15 (IQR 7-28)/10^6^ at baseline versus 17 (7-32)/10^6^ at 12 weeks, p = 0.164).

The only parameter that significantly improved from baseline (3.5 mg/L, IQR 2.1-5.9) to 3 months (2.2, IQR 1.2-4.1, p < 0.001) was high sensitive C-reactive protein.

### Predictors of CIMT progression

The relation from progression in CIMT (ΔCIMT) to potential risk factors was analyzed in a linear multivariate regression model. This model was built on ΔCIMT as the outcome variable and a set of predictor variables. The predictor variables were selected based on univariate regression modelling, with ΔCIMT as outcome-variable. We identified 14 variables with a p-value below 0.2 in the univariate model, which were selected for the final multifactorial regression model. Additionally, the variables sex and age were included as potential confounder-variables for model adjustment as well as statin treatment at baseline and 3 months. The linear multivariate regression model did not yield statistical significance for CIMT prediction at two years (p = 0.16) (Table 4).

**Table 4:** Model-coefficients of predictor variable of the linear multivariate regression model with ΔCIMT as outcome variable (BMI was excluded because of high-correlation with Waist)

	β^	**95% CI**	**p-value**
Waist circumference (Baseline)	0.19	(-0.05 to 0.44)	0.12
HDL-cholesterol (Baseline)	0.10	(-0.26 to 0.46)	0.56
Triglycerides (Baseline)	-0.02	(-0.06 to 0.02)	0.33
Serum creatinine (Baseline)	-1.32	(-15.08 to 12.45)	0.85
Smoking (Baseline)	-4.15	(-12.53 to 4.22)	0.32
Blood glucose 2h (Baseline)	0.00	(-0.04 to 0.04)	0.98
CHD risk score (Baseline)	-0.25	(-0.76 to 0.26)	0.33
PROCAM risk score (Baseline)	0.20	(-0.21to 0.6)	0.34
Statin therapy (Baseline)	-3.95	(-10.78 to 2.87)	0.25
CIMT (Baseline)	-11.29	(-37.99 to 15.4)	0.40
Δ HDL-cholesterol	-0.02	(-0.15to 0.12)	0.82
Δ Triglycerides	0.00	(-0.05 to 0.05)	0.99
Δ NTproBNP	-0.02	(-0.04 to 0)	0.09
Δ Blood glucose 2h	0.04	(-0.11 to 0.2)	0.58
Sex	-1.30	(-11.66 to9.06)	0.80
Age (Baseline)	0.02	(-0.69 to 0.72)	0.96
Statin therapy (3 Months)	-3.86	(-10.8 to 3.08)	0.27

CHD = Coronary Heart Disease (UKPDS risk engine 2.0); PROCAM – Prospective Cardiovascular Münster; IMT – Intima Media Thickness; NTproBNP = N-terminal Pro B-Type Natriuretic Peptide; β^ = estimates of beta coefficient; CI = confidence interval.

Male gender, statin therapy and smoking were assigned a value of 1. Female gender, no statin therapy and non-smoking were assigned a value of 0.

## Discussions

Our study demonstrated that an intensive multifactorial risk factor intervention over 3 months in a specialized diabetes center can lead to sustainably improved risk factor control and a regression of carotid intima media thickness over the period of 2 years. Neither established risk factors nor endothelial function or other novel biomarkers and their changes during a 3 months period of intensified risk factor management were able to explain changes of carotid atherosclerosis.

The STENO-2 study was a trial investigating multifactorial interventions, targeting hypertension, hyperglycaemia and hyperlipidemia which impressively demonstrated the superiority of an intensive risk factor management as compared to a less intensive approach with respect to non-fatal and fatal cardiovascular event reduction [2,16]. Despite the fact that intensive risk factor control deteriorated somewhat after the first 3 months in our study, 24.4% of participants achieved an HbA_1c_ target of <6.5% (<48 mmol/mol), 72.9% an LDL-C target of <2.6 mmol/l and 58.5% a systolic blood pressure <130 mmHg at two years, representing higher percentages for systolic blood pressure and HbA_1c_ target achievement than in the STENO-2 study or the recently published interim analysis of the MIND.IT study, a cluster randomized trial evaluating the feasibility and effectiveness of intensive multifactorial risk factor intervention in T2DM [17].

CIMT is an independent predictor of future cardiovascular events and is often used as a surrogate marker for the presence of cardiovascular disease [18] and future vascular events [19]. Baldassarre et al. have recently shown that fast progression over a 15 months period was associated with an increased subsequent vascular event risk [5].

They investigated subjects with at least 3 cardiovascular risk factors and have observed a progression in all CIMT measurements over 15 months. A sub-study of the Outcome Reduction with an Initial Glargine Intervention (ORIGIN) trial investigated the effect of insulin glargine and n-3 fatty acids on carotid IMT [20]. The CIMT at baseline in the ORIGIN study was 0.88 ± 0.25 mm and therefore similar to the 0.883 ± 0.120 mm observed in our study. Although there was a trend towards a somewhat reduced IMT progression rate in the glargine treated group, it did not reach statistical significance. Of note, the annual CIMT progression observed in the placebo group was 0.026 ± 0.002 mm (maximum CIMT for 12 carotid artery segments). Analyses from lipid and blood pressure lowering trials, which included only a small number of subjects with diabetes, showed somewhat lower annual progression rates (0.015 ± 0.053 mm) [21].

When interpreting our study results, one needs to keep in mind that, as observed in the ORIGIN sub-study the natural course would have been a mean progression of about 0.050 mm (i.e. 5.6%) in the overall cohort over these 2 years rather than a 2.6% regression as observed in our study.

Previous studies demonstrated that single risk factor intervention like glucose lowering [22], lipid lowering [23] or blood pressure lowering [24] can delay CIMT, but to the best of our knowledge this is the first prospective study showing the impact of multifactorial risk factor intervention on CIMT in subjects with type 2 diabetes. CIMT, in our cohort, was significantly associated with age (p = 0.012), duration of diabetes (p < 0.001) as well as systolic blood pressure (p = 0.001).

Endothelial function as measured by the EndoPAT technique was not associated with the overall cardiovascular risk and did not improve with multifactorial risk factor intervention. Previous studies in patients with type 2 diabetes mellitus have also shown conflicting results with regard to endothelial function using statins [25] or blood pressure lowering medication [26]. However, it has been questioned whether the EndoPAT measurements are directly comparable to other assessments of endothelial dysfunction, like flow mediated dilation, since hyperaemia is captured by finger probes as compared to vasodilation assessment of larger arteries [27].

We assessed further markers of endothelial function like ADMA and SDMA, which both turned out not to improve by the intensified treatment or to be of any help regarding the prediction of atherosclerosis progression in our cohort.

Although a previous study in 28 subjects demonstrated an increase of EPCs by multifactorial treatment in patients with type 2 diabetes as assessed by an in-vitro culture assay [12], we were not able to reproduce this finding when measuring CD133/VEGF-R2+ cells by FACS analysis.

With the intention of a personalized treatment approach there is an urgent need to identify novel biomarkers for cardiovascular risk prediction in subjects with diabetes. Since currently a large number of outcome trials is ongoing [28] of which the majority also collects biomarker samples, we expect a plethora of opportunities for targeted and untargeted biomarker research initiatives in this field within the next years.

### Strengths and limitations

The major strengths of our trial are that we included a representative group of subjects with type 2 diabetes without cardiovascular events and that we were able to achieve currently recommend treatment targets in more patients than in other studies (STENO-2, MIND.IT) and the follow-up period of two 2 years for the primary outcome measurement chosen is reasonably long.

One limitation of our study is the uncontrolled design of our trial. However, the aim of this trial was to assess CIMT progression in subjects with type 2 diabetes treated according to current guidelines and to possibly identify early markers predictive of atherosclerosis progression rather than comparing different treatment targets in these subjects. In addition it would have been unethical to recommend less stringent treatment targets to a control group than currently suggested by treatment guidelines.

Our baseline CIMT measurement is similar to the one of the ORIGIN sub-study. Therefore the placebo group of the ORIGIN trial could be adopted as a control group, demonstrating the natural course of CIMT in subjects with dysglycaemia over time and helps putting our results into perspective.

Another limitation is that although CIMT is associated with risk for future cardiovascular events, previous studies have shown that adding CIMT to established risk calculators does not improve risk prediction [29]. In this study we did not investigate the additional predictive value of CIMT for cardiovascular events but the change of CIMT by intensified risk factor treatment. Spence et al. have previously suggested that the extend of risk factor treatment should be determined by atherosclerosis progression rate rather than by the same treatment target for all subjects, however, this approach needs to be tested in a dedicated trial [30].

While CIMT is a measure of early atherosclerosis, carotid plaques reflect a more advanced stage of the disease [31]. Despite a correlation between CIMT and plaque volume measurements and the fact that both are predictors of cardiovascular events, they are not interchangeable and are determined by different risk factor patterns [32-35]. For this study we have chosen CIMT because of the advantage of standardization of measurement and the plethora of randomized controlled trials using this surrogate marker [21].

In summary we were able to show an overall regression of mean CIMT by multifactorial risk factor intervention in subjects with T2DM. However, neither baseline characteristics nor changes during a 3 months intensified treatment period of established risk factors, endothelial function marker, endothelial progenitor cells or inflammation were able to predict changes in the progression of carotid atherosclerosis. Our data suggest that besides novel, yet to be identified biomarkers, changes in CIMT might be used as a measure of treatment response and could help identify subjects with high residual cardiovascular risk despite guideline compliant treatment but this approach would need to be tested in future trials.

## Competing interests

The authors declare that they have no competing interests.

## Authors’ contributions

HS and TCW wrote the study protocol and designed the study. HS oversaw the conduct of the study. NJT and SHN carried out the statistical analysis and NJT and HS wrote the manuscript. ME and NJT acquired data. TCW and TRP contributed to the data interpretation and reviewed and edited the manuscript. All authors read and approved the final manuscript.
